# Factors Associated with Antimicrobial Use in Fijian Livestock Farms

**DOI:** 10.3390/antibiotics11050587

**Published:** 2022-04-27

**Authors:** Xavier Khan, Caroline Rymer, Rosemary Lim, Partha Ray

**Affiliations:** 1 Department of Animal Sciences, School of Agriculture, Policy and Development, University of Reading, Reading RG6 6EU, UK; c.rymer@reading.ac.uk (C.R.); partha.ray@TNC.ORG (P.R.); 2 Reading School of Pharmacy, School of Chemistry, Food & Pharmacy, University of Reading, Reading RG6 6DZ, UK; r.h.m.lim@reading.ac.uk; 3 The Nature Conservancy, 4245 North Fairfax Drive, Suite 100, Arlington, VA 22203, USA

**Keywords:** antimicrobial use, antimicrobial resistance, farm factors, farmer factors, livestock farms, livestock enterprises, socio-economic, demographic, Fiji

## Abstract

Antimicrobial stewardship (AMS) programmes in human health and livestock production are vital to tackling antimicrobial resistance (AMR). Data on antimicrobial use (AMU), resistance, and drivers for AMU in livestock are needed to inform AMS efforts. However, such data are limited in Fiji. Therefore, this study aimed to evaluate the association between farmer (socio-economic, demographic) and livestock production and management factors with AMU. Information was collected using purposive and snowball sampling from 236 livestock farmers and managers located in Central and Western divisions, Viti Levu, Fiji. Multinomial logistic regression was used to determine the factors associated with AMU in farms using an aggregated livestock farm model. Farms that raised cattle only for dairy (farm factor) were more likely to use antibiotics and anthelmintics (*p* = 0.018, OR = 22.97, CI 1.713, 308.075) compared to mixed cattle and poultry farms. Farms that maintained AMU records were more likely to use antibiotics (*p* = 0.045, OR = 2.65, CI 1.024, 6.877) compared to farms that did not. Other livestock production and management factors had no influence on AMU on the livestock farms. AMU in livestock farms was not influenced by the socio-economic and demographic characteristics of the farmer. There were differences between livestock enterprises regarding their management. The lack of association between management system and AMU could be because there was so much variation in management system, levels of farmer knowledge and awareness of AMU, and in management of farm biosecurity. Future studies exploring farmers’ knowledge and awareness of AMU and livestock management are required to design AMS programmes promoting prudent AMU in all livestock farms locally.

## 1. Introduction

Antimicrobial resistance (AMR) is a major global threat to human and animal health [1,2,3]. International collaborative efforts by the World Organisation of Animal Health (OIE), the World Health Organization (WHO) and the Food and Agricultural Organization of the United Nations (FAO) have adopted the One Health approach to combat the global risk of AMR [1,2,3]. In doing so, the prudent use of antimicrobials in livestock production systems have been encouraged [1,2]. Additionally, with the increasing risks of transmission of AMR microbes from livestock farms into the environment and agri-food chain, antimicrobial stewardship (AMS) programmes in the human and animal sectors have been advocated [1,2]. Although antimicrobial use (AMU) data are becoming more accessible, the information on drivers of AMU, which are essential in developing AMS programmes, remains unclear.

Socio-economic and demographic factors influence livestock production systems and management practices [4,5,6]. Backyard farmers produce livestock for domestic consumption and at times sell them to buy plant-based food products [7,8]. These backyard and semi-commercial farmers’ management practices are also influenced by other farmers, friends, and neighbours, thus shaping the attitude and intention of the farmers [9,10]. Socio-economic status may also affect farmers’ ability to seek veterinary advice on animal health, production and improve farm biosecurity infrastructure [7,11,12,13]. In principle, veterinary services affect farmers’ decision-making process, since veterinarians may serve as farmers’ knowledge hub [14]. The advice disseminated by veterinarians to farmers also shapes the behaviour of the farmers [15,16].

Fiji is a developing tropical country with backyard and semi-commercial enterprises such as beef, dairy, chickens, and laying hens, predominantly providing food and financial security to many Fijian farmers [17,18]. Shortages in veterinary professionals have been reported in the livestock sector [19] and are similar to other developing countries [20]. In the human health sector, AMR has been reported [19], but AMR in livestock is unknown. Prudent use of antimicrobials has been advocated at global levels using AMS programmes [1,2,3]. However, implementing mitigation policies surrounding prudent AMU in developing countries such as Fiji is challenging, noting the vast difference in livestock production, management practices, socio-economic and demographic factors [6,20,21]. In developing countries, antimicrobials have been used prophylactically and to increase production [22,23]. Additionally, a lack of knowledge and understanding of AMU and AMR have also been reported [13,24,25].

**Figure 1:**
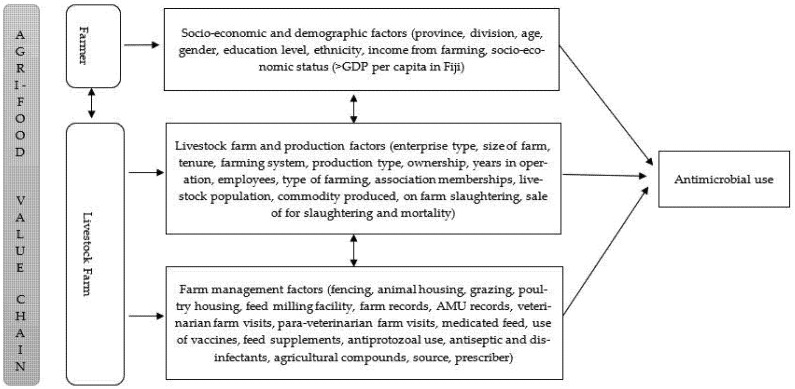
The conceptual framework illustrating the overarching constructs influencing antimicrobial use.

Contextual and socio-psychological factors influence farmers′ attitudes towards farm biosecurity risk management and AMU practice [6,14,26]. However, farm biosecurity risk management strategies differ between farm enterprises [27,28]. Antimicrobials (antibiotics and anthelmintics), nutraceuticals and other medicinal products have been used in livestock production to manage and mitigate farm biosecurity risks [28,29,30]. Nevertheless, the use of these medicinal and non-medicinal products is substantially different in poultry and cattle [29,31]. For instance, antibiotics are administered in flocks of chickens compared to individual animals in cattle herds [31,32,33]. The information about on-farm biosecurity risk management using medicinal and non-medicinal products, including antimicrobials and effects of contextual drivers on AMU practice in Fijian livestock farms, is largely unknown. Our earlier studies have demonstrated the AMU and the patterns of use and lack of knowledge and understanding of AMU and AMR among farmers and para-veterinarians; however, the contextual farmer and farm factors driving AMU remain unexplained. It was hypothesized that farmers′ socio-economic and demographic factors and livestock production and management characteristics influenced the AMU, illustrated in the conceptual framework (see Figure 1). Therefore, this study aimed to investigate the agri-food value chain factors (farmer and livestock farm production and management) that influence AMU in the Central and Western regions of Viti Levu, Fiji.

## 2. Results

The summary of statistically significant farmer and farm factors associated with AMU are presented in Table 1 (farm model). Refer to Appendix A Table A1 (farm model) for a detailed description of all factors.

### 2.1. Livestock Farm Model

#### 2.1.1. Characteristics of Fijian Livestock Farmers and Farms

Table A1 shows the characteristics of the 236 livestock farmers and farms. Most participants were farmers (*n* = 211, 89%) and were from the Western Division of Viti Levu (*n* = 143, 61%). The majority were from Ba province (*n* = 84%) and were 40–59 years of age (*n* = 120, 51%). Most farmers were male (*n* = 198, 84%) and had obtained secondary education qualifications (*n* = 142, 60%). Most farmers reported their income from farming comprised between 25–50% of total household income (*n* = 94, 40%), and their household income was less than gross domestic income per capita (*n* = 151, 64%). Most respondents were not members of any associations (*n* = 176, 75%). Most farms were household-owned (*n* = 162, 69%) with Itaukei Land Trust Board (TLTB) leased tenure (*n* = 63, 27%). Most farms were medium–large holders with farm sizes greater than 2 ha (*n* = 185, 78%) raising livestock in semi-commercial farming systems (*n* = 144, 61%) and classified as organic (*n* = 101, 43%). Most farms were not mixed (crop and livestock) and raised livestock only (*n* = 162, 69%) and were in operation for more than 10 years (*n* = 101, 43%). Most farms employed no farmworkers (*n* = 134, 57%). The most numerous enterprises were beef, which comprised the only livestock on the farm (*n* = 57, 24%). Most farms were small–medium sized herds or flocks (*n* = 171, 72%). Most farms were fenced (*n* = 133, 56%) and had an animal house (*n* = 150, 64%). Half the farms had no para-veterinarian farm visits (*n* = 118, 50%), and the vast majority had no veterinarian visits (*n* = 223, 94%). Although most farmers had maintained farm records (*n* = 122, 52%), very few farms maintained AMU records (*n* = 38, 16%). Most farms had no on-farm milling facility (*n* = 220, 93%) and had not used medicated feed (*n* = 125, 53%). Most farms had not used any feed supplements (*n* = 202, 86%). Most farms had not used antiprotozoal (*n* = 229, 97%) or herbal preparations (*n* = 211, 89%). However, most had used vitamins and minerals (*n* = 122, 52%). Very few farms had used vaccines (*n* = 11, 5%), and the majority of farms had also not used antiseptics and disinfectants (*n* = 193, 82%) or other agricultural compounds (herbicides and pesticides) (*n* = 232, 98%).

#### 2.1.2. Livestock Farm Modelling

Of the 34 variables presented in Table A1, only 15 variables (division, province, gender, association memberships, farm size, years in operations, enterprise type, fencing, animal housing, para-veterinarian farm visits, veterinarian farm visits, AMU records, medicated feed use, feed supplement use, antiseptics and disinfectants use) were associated with AMU (*p* < 0.05) (see Table 1). Farms that raised cattle only for dairy were more likely to use antibiotics and anthelmintics (*p* = 0.018, OR = 22.97, CI 1.713, 308.075). Dairy farms were more likely to use antibiotics only (*p* = 0.097) and anthelmintics only (*p* = 0.594). There was a tendency (*p* = 0.848) for beef-only farms to use both anthelmintics and antibiotics. Farms which had both a beef and dairy enterprise used both antibiotics and anthelmintics (*p* = 0.467). The layer-only (*p* = 0.917), broiler-only (*p* = 0.356), and layer and broiler mixed farms (*p* = 0.698) were most likely to use antibiotics. Smallholder farms were less likely to use a combination of both (*p* = 0.015 OR = 0.15, CI 0.032, 0.689).

Interestingly, farms that maintained AMU records were more likely to use antibiotics ( *p*= 0.045, OR = 2.65, CI 1.024, 6.877) and similarly anthelmintics only (*p* > 0.05). Farms that had not used medicated feeds were more likely to use anthelmintics only (*p* < 0.001, OR = 11.56, CI 3.456,38.604) and a combination of both (anthelmintics and antibiotics) (*p* = 0.017, OR =3.10, CI 1.222, 7.882). Farms that had not used feed supplements were also more likely to use anthelmintics only (*p* = 0.025, OR = 6.37, CI 1.261, 32.155) or both (*p* = <0.001, OR = 30.41, CI 7.277, 127.081). In contrast, farms that had not used antiseptics and disinfectants were less likely to use antimicrobials (see Table 2).

## 3. Discussion

This study, to our knowledge, is the first study investigating the factors associated with AMU in Fijian livestock farms. Our study demonstrated that AMU in livestock farms was not influenced by the socio-economic and demographic characteristics of the farmer, but influenced by the livestock production and management factors such as species of farmed livestock (enterprise type), and farm management factors (AMU records, medicated feed use and feed supplements) (see Table 2). Despite the differences in farming systems and management practices, most factors were not associated with AMU. Other studies have demonstrated the effects of livestock management and farm biosecurity systems on AMU practice [27,28]. Given that seasonal conditions affect disease burdens such as helminthic and bacterial infections in different livestock enterprises; therefore, the chances of using a type of antimicrobial could be higher. However, the AMU practices in the livestock farms surveyed differed and we could not explain a lack of association between farm factors and AMU [34]. Other studies have demonstrated that socio-economic and demographic factors influence AMU practice [7,14,35], and this may have been the case in our study as well, but we were unable to detect it because of an unequal representation of farmers from all socio-economic and demographic groups and farming systems. Hence, we suggest future studies to consider the sampling strategy to ensure equal representation and larger sample size so that modelling could be executed to better predict the AMU practice in different enterprises and systems.

As flock-level administration of antimicrobials in poultry is more likely compared to an individual animal in cattle [32,33,36], the chances of antimicrobial use in specialist cattle and poultry and mixed cattle and mixed poultry were higher than other mixes of farm enterprises [6,31]. We believe the higher incidence of mammary infections in dairy cows may be the reason for the increased use of antibiotics in cattle [34]; however, we also believe the use may be for prophylaxis and growth promotion, as demonstrated in our previous study [37]. The chances of antibiotic use were higher in poultry enterprises due to flock-level administration, prophylaxis and growth promotion, as demonstrated in our previous study [38]. Our finding of higher chances of using antimicrobials in cattle and poultry enterprises is similar to findings demonstrated in other studies [31,36]. Although flock/herd density is higher in commercial systems than semi-commercial and backyard, there was no influence of the farming system on AMU (*p* = 0.430). Our earlier study also demonstrated that the farming system did not affect AMU when quantified using different metrics [38]. However, we also believe this similar use between farming systems may be due to a lack of knowledge and understanding of AMU, leading in some cases to an unwitting use of antimicrobials. We could not establish statistical significance between farming systems factors due to modelling inefficiencies. We believe that lower chances of AMU in smallholder farms may be due to a lack of access to antimicrobials [39]. The association between maintenance of AMU records and antibiotic use may be a consequence of farmers producing poultry to contract being required to provide records of AMU to commercial processors [40]. Farmers that were not producing for a contract may be using antimicrobials but have not kept records of AMU because of a lack of knowledge and understanding of the importance of maintaining such records. This has been demonstrated in earlier studies [39,40]. Our earlier study demonstrated that Fijian farmers lacked general knowledge and understanding of medicines and did not differentiate between different types of medicine [39]. We believe farmers considered medicated feed, feed supplements, antiseptics, and disinfectants also as medicines and used them on their livestock [39]. We believe farmers use medicated feed (containing antimicrobials) and feed supplements to prevent animal diseases and promote growth [41] and used antimicrobials as the first line of treatment [37,39]. It was beyond the scope of the current study to explain the motivations behind the use of other medicines (vaccines, topical antimicrobials, antiprotozoals, multivitamins and minerals, feed supplements, herbal preparations, antiseptics and disinfectants and agricultural compounds). Therefore, further studies investigating the drivers of other medicines used are required, so that other medicines used including AMU in Fijian livestock production systems could be better understood.

Our study revealed that farmers used antibiotics, anthelmintics, commercial feed, nutraceuticals, herbal medicines, and vaccines, and these may have been used to mitigate farm biosecurity risks or for other purposes [28,29,42]. Farm biosecurity infrastructure was in place in most farms, but farmers with lower socio-economic status do find it challenging to implement farm biosecurity risk mitigation measures because of the associated costs [14,22,42,43,44]. Nonetheless, our studies showed no association with farm infrastructure factors in most farms.

Maintenance of farm records is another essential part of farm biosecurity assessment [44]. Although most farmers had attained secondary school education, most farms did not maintain farm AMU records. We do not believe that literacy was an issue but understanding the importance of maintaining farm AMU records may be an essential consideration. Hence, we suggest follow up studies exploring attitude and knowledge towards record-keeping and overall biosecurity risk management on farms. Our study revealed that level of education was not associated with AMU practice, but exploring the knowledge and understanding of farmers on AMU and AMR at the enterprise level may be an essential consideration as reported in studies in other settings [24,39]. Additionally, this may inform and assist in developing mitigation strategies adopted as part of AMS programmes.

Veterinarians and para-veterinarians have a critical role in AMS programmes [45], but our study revealed veterinarian and para-veterinarian farm visits were very low. Interaction between farmers with veterinarians and para-veterinarians is therefore low, resulting in imprudent AMU practices as farmers self-prescribe antimicrobials [24,26,39,46]. Also, this may provide a window of opportunity to farmers who may opt to explore other avenues for advice, as is the case in other countries [47]. It must be noted that technical and clinical guidance on managing animal health and farm risks offered by veterinarians are more informed and cannot be compared to other sources [14]; therefore, it is imperative that improving Fijian veterinary services should be considered and incorporated as a critical priority indicator when developing policies in AMS programme so that better farm risk management practices are implemented. Self-prescribing is a common problem in the human health sector; thus, the chances of the same behaviour adopted by farmers in livestock farms is of grave concern as also indicated in our previous studies [37,39,39]; hence such practices need to be further explored [48]. We, therefore, recommend further studies exploring self-prescribing behaviour patterns of farmers so that more informed behavioural change intervention could be recommended.

Although participants were unequally represented by gender, our findings are not extraordinary since farm ownership, and farm decisions are traditionally made by the head of household, usually male, consistent with findings in other studies [49]. Nevertheless, the literature review informed our hypothesized conceptual framework (Figure 1), which assisted in elucidating valuable information about Fijian livestock production and management practices; AMU practices, the nutraceuticals and herbal medicines used, and the feed and feeding systems which were unknown. Therefore, the conceptual framework provided can be used to elucidate information on livestock production systems in other developing countries like Fiji, where information is limited.

## 4. Materials and Methods

### 4.1. Study Design and Data Collection

Data on farmer and farm characteristics, livestock production, feed and feeding practices and medicine use, collected from the cross-sectional survey conducted between May to August 2019 in Central and Western Divisions of Viti Levu, Fiji, previously reported in Khan et al. (2021) [38] was evaluated in this present study. Purposive and snowball sampling was used to recruit farmers and farm managers. A total of 236 livestock farms were investigated [38]. Considering the farmer and livestock farm constructs in the conceptual framework (see Figure 1), factors associated with AMU were assessed using the aggregated livestock farm model (see Figure 1 and Table A1 (farm model) for a detailed description of all factors).

### 4.2. Data Management and Analysis

Data were analysed using IBM SPSS Software V27. Farmer characteristics (socioeconomic and demographic), livestock farm production and livestock management, including feed and feeding practices, medicinal and non-medicinal product use, and antimicrobial access variables (factors) (see Figure 1 and Table A1 (farm model) for a detailed description of all factors), were descriptively analysed. Frequency and percentages were summarised for categorical factors. Data on other medicines used, excluding antimicrobials administered orally, parenterally and intramammary, were assessed and classified into vaccines, topical antimicrobials, antiprotozoals, multivitamins and minerals, feed supplements, herbal preparations, antiseptics and disinfectants and agricultural compounds [29]. These other categories of medicines used were coded (either used or not used). Continuous factors were reclassified into categories. These were: years of operation (<5 years, 5–10 years, >10 years) [12], number of employees (0, 1, ≥2), farm size (smallholder farm ≤2 hectares (ha), medium–large ≥2 ha) [50], para-veterinary and veterinary visits (no visits, monthly, quarterly) and herd/flock size (as reported in an earlier study [38]) was classified into three categories based on farming system (backyard, semi-commercial = small–medium, commercial = large) [51,52]. From the antimicrobial use data, outcome factor (AMU) was categorised into types of antimicrobial used (antibiotics, anthelmintics, both and none) [38]. Our earlier study demonstrated that antimicrobials were mainly sourced from veterinary clinics and self-prescribed by farmers; hence factors (source and prescriber of antimicrobial) were excluded in this study [37]. A total of 34 variables for the livestock farm model were considered for analysis (see Figure 1 and Table A1 (farm model) for a detailed description of all factors).

### 4.3. Statistical Analysis

A livestock farm model was developed using the livestock farm data (see Figure 1 for a detailed description of all factors). Chi-square test or Fisher′s exact test as appropriate, were used to investigate the association between hypothesized independent factors (farmer characteristics, farm production and management characteristics) with outcome factor (AMU) [53,54]. Statistically significant independent factors were fitted into multinomial logistic regression models to investigate the relationship between the independent factors and AMU [30,55]. The independent factors with *p* < 0.2 in univariate analysis were retained, and model reduction was done manually with confounding factors eliminated from the model [56,57]. The ‘no AMU′ outcome category was set as a reference category in livestock farm modelling. Odds ratios with 95% confidence intervals (95% CI) were reported, and *p* < 0.05 was considered statistically significant.

## 5. Conclusions

This study suggests that the AMU on livestock farms was not influenced by livestock farmers′ socio-economic and demographic characteristics. AMU was more likely in cattle (dairy) farms, and antibiotic use in poultry (broiler, layer and broiler) farms, compared to mixed cattle and poultry farms. Future studies exploring knowledge and awareness of AMU and livestock management, including farm biosecurity risk management, are required to design and implement AMS programmes to promote prudent AMU in Fijian livestock production systems. Further studies exploring the social and cultural factors driving the AMU are required to better understand the drivers of AMU practice at the national level.

## Figures and Tables

**Table 1:** Summary of associations between factors (farmer, livestock production and management) and antimicrobial use (antibiotics, anthelmintics, both and no antimicrobial use) on 236 livestock farms located in Central and Western divisions of Viti Levu, Fiji.

Factor	Total		Antimicrobial Use								*p* Value
			Antibiotics		Anthelmintics		Both		No AMU		
	n	(%)	n	(%)	n	(%)	n	(%)	n	(%)	
Division											
Central	93	(39)	27	(47)	12	(33)	25	(52)	29	(31)	0.038
Western	143	(61)	30	(53)	24	(67)	23	(48)	66	(69)	
Province											
Naitasiri	26	(11)	8	(14)	6	(17)	7	(15)	5	(5)	0.001
Namosi	13	(6)	2	(4)	1	(3)	2	(4)	8	(8)	
Rewa	13	(6)	5	(9)	1	(3)	0	(0)	7	(7)	
Serua	19	(8)	5	(9)	4	(11)	6	(13)	4	(4)	
Tailevu	22	(9)	7	(12)	0	(0)	10	(21)	5	(5)	
Ba	84	(36)	15	(26)	14	(39)	21	(44)	34	(36)	
Nadroga-Navosa	28	(12)	5	(9)	8	(22)	2	(4)	13	(14)	
Ra	31	(13)	10	(18)	2	(6)	0	(0)	19	(20)	
Gender											
Male	198	(84)	48	(84)	34	(94)	44	(92)	72	(76)	0.021
Female	38	(16)	9	(16)	2	(6)	4	(8)	23	(24)	
Association memberships											
Yes	60	(25)	10	(18)	14	(39)	22	(46)	14	(15)	<0.001
No	176	(75)	47	(82)	22	(61)	26	(54)	81	(85)	
Farm size											
Small holder (<2 ha)	51	(22)	14	(25)	2	(6)	3	(6)	32	(34)	<0.001
Medium-large holder (>2 ha)	185	(78)	43	(75)	34	(94)	45	(94)	63	(66)	
Years in operation											
<5 years	67	(28)	19	(33)	4	(11)	5	(10)	39	(41)	<0.001
5–10 years	68	(29)	17	(30)	8	(22)	15	(31)	28	(29)	
>10 years	101	(43)	21	(37)	24	(67)	28	(58)	28	(29)	
Fencing											
Yes	133	(56)	28	(49)	24	(67)	36	(75)	45	(47)	0.005
No	103	(44)	29	(51)	12	(33)	12	(25)	50	(53)	
Enterprise type											
Beef only	57	(24)	10	(18)	17	(47)	8	(17)	22	(23)	<0.001
Dairy only	52	(22)	9	(16)	11	(31)	29	(60)	3	(3)	
Beef and dairy	11	(5)	0	(0)	2	(6)	4	(8)	5	(5)	
Layer only	50	(21)	13	(23)	3	(8)	2	(4)	32	(34)	
Broiler only	38	(16)	18	(32)	0	(0)	1	(2)	19	(20)	
Layer and broiler	12	(5)	4	(7)	0	(0)	1	(2)	7	(7)	
Mixed cattle and poultry	16	(7)	3	(5)	3	(8)	3	(6)	7	(7)	
Animal housing											
Yes	150	(64)	43	(75)	13	(36)	22	(46)	72	(76)	<0.001
No	86	(36)	14	(25)	23	(64)	26	(54)	23	(24)	
Para-veterinarians farm visits											
No visits	118	(50)	21	(37)	14	(39)	20	(42)	63	(66)	0.004
quarterly	74	(31)	20	(35)	15	(42)	19	(40)	20	(21)	
monthly	44	(19)	16	(28)	7	(19)	9	(19)	12	(13)	
Veterinarian farm visits											
No visits	223	(94)	46	(81)	35	(97)	48	(100)	94	(99)	<0.001
quarterly	4	(2)	2	(4)	1	(3)	0	(0)	1	(1)	
monthly	9	(4)	9	(16)	0	(0)	0	(0)	0	(0)	
AMU records											
Yes	38	(16)	16	(28)	8	(22)	4	(8)	10	(11)	0.010
No	198	(84)	41	(72)	28	(78)	44	(92)	85	(89)	
Medicated feed used											
Not used	125	(53)	22	(39)	32	(89)	35	(73)	36	(38)	<0.001
Used	111	(47)	35	(61)	4	(11)	13	(27)	59	(62)	
Feed supplements											
Not used	202	(86)	53	(93)	30	(83)	27	(56)	92	(97)	<0.001
Used	34	(14)	4	(7)	6	(17)	21	(44)	3	(3)	
Antiseptics and disinfectants											
Not used	193	(82)	44	(77)	30	(83)	31	(65)	88	(93)	<0.001
Used	43	(18)	13	(23)	6	(17)	17	(35)	7	(7)	

Note: Zero (0) indicates no participant in that category, *n* denotes frequency, and % denotes percentage observed, both denotes antibiotics and anthelmintics were used, AMU denotes antimicrobials used. *p*-value denotes the probability of association obtained using the Chi-square test or Fisher′s exact test as appropriate between antimicrobial use (antibiotic, anthelmintic, both and no AMU) and factors (farmer, livestock production and management).

**Table 2:** Multinomial logistic modelling analysis of factors (farmer, livestock production and management) with antimicrobial use on 236 livestock farms located in Central and Western divisions of Viti Levu, Fiji.

Factor	Antimicrobial Use					
	Antibiotics		Anthelmintics		Both	
	*p* Value	OR (95% CI)	*p* Value	OR (95% CI)	*p* Value	OR (95% CI)
Enterprise type						
Beef only	0.934	0.92 (0.131, 6.456)	0.428	0.43 (0.053, 3.472)	0.848	1.27 (0.114, 13.985)
Dairy only	0.097	6.67 (0.711, 62.490)	0.594	1.91 (0.176, 20.776)	0.018	22.97 (1.713, 308.075)
Beef and dairy	0	0 (0)	0.264	0.23 (0.017, 3.064)	0.467	2.72 (0.184, 40.117)
Layer only	0.917	1.09 (0.227, 5.206)	0.151	0.23 (0.030, 1.719)	0.217	0.27 (0.032, 2.177)
Broiler only	0.356	2.10 (0.434, 10.154)	0	0 (0)	0.057	0.08 (0.005, 1.080)
Layer and broiler	0.698	1.46 (0.217, 9.821)	0	0 (0)	0.541	0.43 (0.029, 6.356)
Mixed cattle and poultry	Ref		Ref		Ref	
Farm size						
Small holder (<2 ha)	0.284	0.64 (0.282, 1.449)	0.104	0.26 (0.050, 1.319)	0.015	0.15 (0.032, 0.689)
Medium-large holder (>2 ha)	Ref		Ref		Ref	
AMU records						
Yes	0.045	2.65 (1.024, 6.877)	0.051	3.48 (0.993, 12.166)	0.406	0.53 (0.120, 2.354)
No	Ref		Ref		Ref	
Medicated feed used						
Not used	0.894	1.05 (0.496, 2.234)	<0.001	11.56 (3.456, 38.604)	0.017	3.10 (1.222, 7.882)
Used	Ref		Ref		Ref	
Feed supplements						
Not used	0.247	2.52 (0.527, 12.003)	0.025	6.37 (1.261, 32.155)	<0.001	30.41 (7.277, 127.081)
Used	Ref		Ref		Ref	
Antiseptics and disinfectants						
Not used	0.076	0.39 (0.136, 1.105)	0.283	0.49 (0.136, 1.789)	0.001	0.15 (0.047, 0.456)
Used	Ref		Ref		Ref	

Note: Zero (0) indicates no participants in that category, *n* denotes frequency, and % denotes percentage observed, both denote (antibiotics and anthelmintics used), AMU denotes antimicrobials used. *p*-value denotes probability for the association, Ref denotes reference group.

## Data Availability

The data presented in this study are available on request from the corresponding author.

## Appendix A

**Table A1:** Associations between factors (farmer, livestock production and management) and antimicrobial use (antibiotics, anthelmintics, both and no antimicrobial use) on 236 livestock farms located in Central and Western divisions of Viti Levu, Fiji.

Factor	Total		Antimicrobial Use								*p* Value
			Antibiotics		Anthelmintics		Both		No AMU		
	n	(%)	n	(%)	n	(%)	n	(%)	n	(%)	
Participant type											
Farmer	211	(89)	51	(89)	35	(97)	44	(92)	81	(85)	0.231
Farm manager	25	(11)	6	(11)	1	(3)	4	(8)	14	(15)	
Division											
Central	93	(39)	27	(47)	12	(33)	25	(52)	29	(31)	0.038
Western	143	(61)	30	(53)	24	(67)	23	(48)	66	(69)	
Province											
Naitasiri	26	(11)	8	(14)	6	(17)	7	(15)	5	(5)	0.001
Namosi	13	(6)	2	(4)	1	(3)	2	(4)	8	(8)	
Rewa	13	(6)	5	(9)	1	(3)	0	(0)	7	(7)	
Serua	19	(8)	5	(9)	4	(11)	6	(13)	4	(4)	
Tailevu	22	(9)	7	(12)	0	(0)	10	(21)	5	(5)	
Ba	84	(36)	15	(26)	14	(39)	21	(44)	34	(36)	
Nadroga-Navosa	28	(12)	5	(9)	8	(22)	2	(4)	13	(14)	
Ra	31	(13)	10	(18)	2	(6)	0	(0)	19	(20)	
Age											
10–19 years	1	(0)	0	(0)	1	(3)	0	(0)	0	(0)	0.293
20–39 years	49	(21)	11	(19)	7	(19)	6	(13)	25	(26)	
40–59 years	120	(51)	27	(47)	20	(56)	27	(56)	46	(48)	
Over 60 years	66	(28)	19	(33)	8	(22)	15	(31)	24	(25)	
Gender											
Male	198	(84)	48	(84)	34	(94)	44	(92)	72	(76)	0.021
Female	38	(16)	9	(16)	2	(6)	4	(8)	23	(24)	
Education level											
Primary	31	(13)	10	(18)	7	(19)	3	(6)	11	(12)	0.850
Secondary	142	(60)	30	(53)	19	(53)	31	(65)	62	(65)	
Tertiary	39	(17)	10	(18)	6	(17)	9	(19)	14	(15)	
Agricultural College	21	(9)	6	(11)	4	(11)	4	(8)	7	(7)	
Never Attended	3	(1)	1	(2)	0	(0)	1	(2)	1	(1)	
Income from farming											
≤25%	71	(30)	13	(23)	11	(31)	12	(25)	35	(37)	0.213
25–50%	94	(40)	21	(37)	12	(33)	21	(44)	40	(42)	
51–75%	30	(13)	10	(18)	8	(22)	6	(13)	6	(6)	
≥76%	41	(17)	13	(23)	5	(14)	9	(19)	14	(15)	
Household income > GDP (Gross Domestic Product) per capita in Fiji											
Yes	85	(36)	23	(40)	14	(39)	19	(40)	29	(31)	0.552
No	151	(64)	34	(60)	22	(61)	29	(60)	66	(69)	
Association memberships											
Yes	60	(25)	10	(18)	14	(39)	22	(46)	14	(15)	<0.001
No	176	(75)	47	(82)	22	(61)	26	(54)	81	(85)	
Farm ownership											
Individual	32	(14)	14	(25)	4	(11)	8	(17)	6	(6)	0.106
Household	162	(69)	30	(53)	27	(75)	33	(69)	72	(76)	
Company	32	(14)	10	(18)	3	(8)	6	(13)	13	(14)	
Cooperative	7	(3)	1	(2)	2	(6)	1	(2)	3	(3)	
Contract farming	3	(1)	2	(4)	0	(0)	0	(0)	1	(1)	
Farm tenure											
Freehold	45	(19)	15	(26)	4	(11)	7	(15)	19	(20)	0.336
Crown Lease	31	(13)	8	(14)	4	(11)	8	(17)	11	(12)	
Agriculture Leased	43	(18)	14	(25)	6	(17)	5	(10)	18	(19)	
TLTB Leased	63	(27)	11	(19)	12	(33)	19	(40)	21	(22)	
Mataqali	44	(19)	8	(14)	8	(22)	6	(13)	22	(23)	
Squatter	2	(1)	0	(0)	1	(3)	1	(2)	0	(0)	
Commercial leased	8	(3)	1	(2)	1	(3)	2	(4)	4	(4)	
Farm size											
Small holder (<2 ha)	51	(22)	14	(25)	2	(6)	3	(6)	32	(34)	<0.001
Medium-large holder (>2 ha)	185	(78)	43	(75)	34	(94)	45	(94)	63	(66)	
Farming systems											
Backyard	27	(11)	8	(14)	2	(6)	3	(6)	14	(15)	0.430
Semi commercial	144	(61)	30	(53)	23	(64)	33	(69)	58	(61)	
Commercial	65	(28)	19	(33)	11	(31)	12	(25)	23	(24)	
Production type											
Organic	101	(43)	24	(42)	14	(39)	21	(44)	42	(44)	0.302
Conventional	70	(30)	22	(39)	7	(19)	13	(27)	28	(29)	
Prefer not to comment	65	(28)	11	(19)	15	(42)	14	(29)	25	(26)	
Farming type											
Livestock only	162	(69)	39	(68)	20	(56)	36	(75)	67	(71)	0.270
Mixed (Crop and Livestock)	74	(31)	18	(32)	16	(44)	12	(25)	28	(29)	
Years in operation											
<5 years	67	(28)	19	(33)	4	(11)	5	(10)	39	(41)	<0.001
5–10 years	68	(29)	17	(30)	8	(22)	15	(31)	28	(29)	
>10 years	101	(43)	21	(37)	24	(67)	28	(58)	28	(29)	
Employees											
0	134	(57)	34	(60)	21	(58)	22	(46)	57	(60)	0.309
<2	25	(11)	7	(12)	5	(14)	8	(17)	5	(5)	
>2	77	(33)	16	(28)	10	(28)	18	(38)	33	(35)	
Enterprise type											
Beef only	57	(24)	10	(18)	17	(47)	8	(17)	57	(24)	<0.001
Dairy only	52	(22)	9	(16)	11	(31)	29	(60)	52	(22)	
Beef and dairy	11	(5)	0	(0)	2	(6)	4	(8)	11	(5)	
Layer only	50	(21)	13	(23)	3	(8)	2	(4)	50	(21)	
Broiler only	38	(16)	18	(32)	0	(0)	1	(2)	38	(16)	
Broiler and layer	12	(5)	4	(7)	0	(0)	1	(2)	12	(5)	
Mixed cattle and poultry	16	(7)	3	(5)	3	(8)	3	(6)	16	(7)	
Flock/herd size											
Small-medium	171	(72)	38	(67)	25	(69)	36	(75)	72	(76)	0.614
Large	65	(28)	19	(33)	11	(31)	12	(25)	23	(24)	
Fencing											
Yes	133	(56)	28	(49)	24	(67)	36	(75)	45	(47)	0.005
No	103	(44)	29	(51)	12	(33)	12	(25)	50	(53)	
Animal housing											
Yes	150	(64)	43	(75)	13	(36)	22	(46)	72	(76)	<0.001
No	86	(36)	14	(25)	23	(64)	26	(54)	23	(24)	
Para-veterinarian farm visits											
No visits	118	(50)	21	(37)	14	(39)	20	(42)	63	(66)	0.004
quarterly	74	(31)	20	(35)	15	(42)	19	(40)	20	(21)	
monthly	44	(19)	16	(28)	7	(19)	9	(19)	12	(13)	
Veterinarian farm visits											
No visits	223	(94)	46	(81)	35	(97)	48	(100)	94	(99)	<0.001
quarterly	4	(2)	2	(4)	1	(3)	0	(0)	1	(1)	
monthly	9	(4)	9	(16)	0	(0)	0	(0)	0	(0)	
Farm records											
Yes	122	(52)	28	(49)	23	(64)	24	(50)	47	(49)	0.469
No	114	(48)	29	(51)	13	(36)	24	(50)	48	(51)	
AMU records											
Yes	38	(16)	16	(28)	8	(22)	4	(8)	10	(11)	0.010
No	198	(84)	41	(72)	28	(78)	44	(92)	85	(89)	
Feed milling on farm											
Yes	16	(7)	1	(2)	3	(8)	3	(6)	9	(9)	
No	220	(93)	56	(98)	33	(92)	45	(94)	86	(91)	0.317
Medicated feed used											
Not used	125	(53)	22	(39)	32	(89)	35	(73)	36	(38)	<0.001
Used	111	(47)	35	(61)	4	(11)	13	(27)	59	(62)	
Feed supplements											
Not used	202	(86)	53	(93)	30	(83)	27	(56)	92	(97)	<0.001
Used	34	(14)	4	(7)	6	(17)	21	(44)	3	(3)	
Antiprotozoal											
Not used	229	(97)	55	(96)	36	(100)	46	(96)	92	(97)	0.703
Used	7	(3)	2	(4)	0	(0)	2	(4)	3	(3)	
Herbal preparations											
Not used	211	(89)	50	(88)	34	(94)	45	(94)	82	(86)	0.384
Used	25	(11)	7	(12)	2	(6)	3	(6)	13	(14)	
Vitamins and minerals											
Not used	114	(48)	33	(58)	14	(39)	19	(40)	48	(51)	0.170
Used	122	(52)	24	(42)	22	(61)	29	(60)	47	(49)	
Vaccines											
Not used	225	(95)	54	(95)	36	(100)	46	(96)	89	(94)	0.490
Used	11	(5)	3	(5)	0	(0)	2	(4)	6	(6)	
Antiseptics and disinfectants											
Not used	193	(82)	44	(77)	30	(83)	31	(65)	88	(93)	<0.001
Used	43	(18)	13	(23)	6	(17)	17	(35)	7	(7)	
Agricultural compounds (herbicides and pesticides)											
Not used	232	(98)	57	(100)	35	(97)	47	(98)	93	(98)	0.711
Used	4	(2)	0	(0)	1	(3)	1	(2)	2	(2)	

Note: zero (0) indicates no participant of that category participated, *n* denotes frequency, and % denotes percentage observed, both denote (antibiotics and anthelmintics used), AMU denotes antimicrobials used and, *p*-value denotes probability of association obtained using Chi-square test or Fisher’s exact test as appropriate between antimicrobial use (antibiotic, anthelmintic, both and no AMU) and factors(farmer, livestock production and management).
